# Printable ink lenses, diffusers, and 2D gratings†
†Electronic supplementary information (ESI) available. See DOI: 10.1039/c6nr07841a


**DOI:** 10.1039/c6nr07841a

**Published:** 2016-11-23

**Authors:** Rajib Ahmed, Ali K. Yetisen, Anthony El Khoury, Haider Butt

**Affiliations:** a Nanotechnology Laboratory , School of Engineering , University of Birmingham , Birmingham B15 2TT , UK . Email: h.butt@bham.ac.uk ; Tel: +44 (0)1214158623; b Harvard Medical School and Wellman Center for Photomedicine , Massachusetts General Hospital , 65 Landsdowne Street , Cambridge , MA 02139 , USA; c Harvard-MIT Division of Health Sciences and Technology , Massachusetts Institute of Technology , Cambridge , MA 02139 , USA

## Abstract

Advances in holography have led to applications including data storage, displays, security labels, and colorimetric sensors. However, existing top-down approaches for the fabrication of holographic devices are complex, expensive, and expertise dependent, limiting their use in practical applications. Here, ink-based holographic devices have been created for a wide range of applications in diffraction optics. A single pulse of a 3.5 ns Nd:YAG laser allowed selective ablation of ink to nanofabricate planar optical devices. The practicality of this method is demonstrated by fabricating ink-based diffraction gratings, 2D holographic patterns, optical diffusers, and Fresnel zone plate (FZP) lenses by using the ink. The fabrication processes were rationally designed using predictive computational modeling and the devices were fabricated within a few minutes demonstrating amenability for large scale printable optics through industrial manufacturing. It is anticipated that ink will be a promising diffraction optical material for the rapid printing of low-cost planar nanophotonic devices.

## Abstract 2

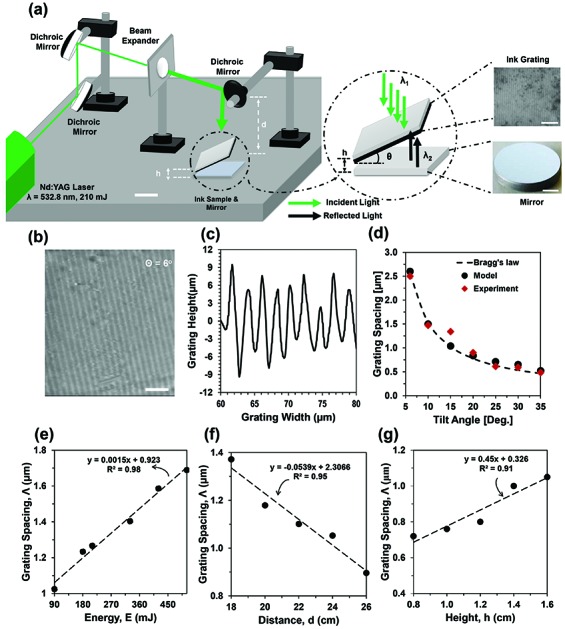
Advances in holography have led to applications including data storage, displays, security labels, and colorimetric sensors.

## 1. Introduction

Holography is a two or three dimensional (2/3D) imaging technique to record and reconstruct amplitude and phase information from photonic nanostructures by means of diffraction.^1^,^2^ High-resolution imaging and multiplexing in holography enable applications in image processing, 3D displays, and security devices.^3^,^4^ Additionally, holographic metasurfaces offer broadband readouts at wide-angles, high-efficiency, and arbitrary wavefront shaping.^5^ However, the fabrication of holographic metasurfaces is complex relying on advanced high-cost manufacturing techniques. Denisyuk reflection holography is a low-cost image recording method that is based on the laser interference between a reference and an object beam that propagates in the opposite direction in a recording medium on a transparent substrate.^6^ Holograms can also be formed on opaque substrates, where the off-axis interference is created by beams traveling toward the same side of the recording medium.^7^ The optical readout of the encoded interference pattern is based on diffraction optics, where the recorded amplitude and phase information are reconstructed at the far field.^2^,^8^

The historical holographic recording has been based on silver halide chemistry, photopolymers, and photoresists to create volumetric or surface holograms.^9^,^10^ Silver-halide based volume holograms are created in a light-sensitive emulsion followed by development and hypo and bleaching stages; however, this approach is limited due to multistep chemical processing. Photopolymer-based volumetric holograms are analogous to silver-halide chemistry, but it requires strict control over the photopolymer properties and stabilization of the interference patterns.^11^ Photoresist-based surface holograms are limited in personalization due to the high cost of preparing the master hologram.^12^ Master surface holograms can be created to emboss rainbow surface holograms; however, volumetric holograms cannot be replicated by this method.^13^ Furthermore, there are also other complex methods including focused ion beam (FIB) milling and electron beam lithography (EBL) to create holograms with nanoscale resolutions, but these methods are limited due to high-cost and time-consuming fabrication processes.^14^–^16^

To overcome the limitations in traditional approaches, direct laser interference pattering (DLIP), which is also known as mask-free lithography has been utilized to create 3D interconnected patterns and off-axis holograms.^17^ The high power laser pulse (300 mJ cm^–2^) allowed for patterning surfaces of polymer, aluminum zinc metal–oxide, TiO_2_, nickel, and steel substrates.^18^–^20^ The optical arrangement of DLIP holography required a complex setup and specific exposure angles of the incident beams for accurate fabrication.^21^ Recently, a single-pulse Denisyuk DLIP holography method was developed to form surface gratings.^7^,^12^ Light absorbing materials such as inks and metal thin films were coated on glass substrates and laser ablation was utilized to selectively remove the material from localized regions.^8^ The precision of the ablated pattern depends on local thermal diffusion in the light-absorbing medium governed by laser intensity, pulse width, and beam wavelength.^7^ This approach created well-ordered parallel surface gratings at low cost. The utilization of single-pulse DLIP holography to create a wide range of 2D and 3D complex geometries is highly desirable for application in nanophotonic devices.

Here, the single-pulse Denisyuk DLIP holography method is demonstrated for creating ink-based planar nanophotonic devices. Ink has been introduced as a recording medium for printing holographic devices. As a demonstration, a single nanosecond laser pulse in Denisyuk reflection mode was used to create diffraction gratings, 2D nanopatterns, optical diffusers, and Fresnel zone plate (FZP) lenses. Ink surface gratings were also demonstrated to tune the radial color focusing of the FZP lens. This high-throughput approach eliminates the need for light-sensitive materials and does not require complex nanofabrication and clean room facilities for surface patterning. Therefore, the utilization of ink as the recording medium reduces the steps for holographic image writing without requiring any chemical processing to save time and reduce costs.

## 2. Experiments and results

### 2.1. Ink-based holographic recording

**Fig. 1:**
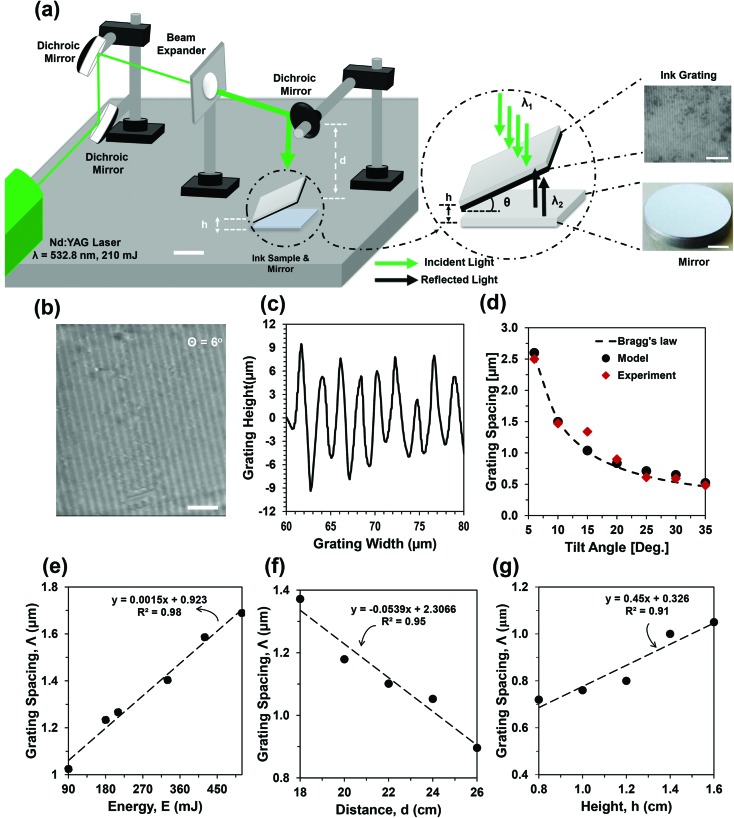
Fabrication of the ink-based surface gratings using Denisyuk reflection holography. (a) The experimental setup for recording ink-based diffraction gratings. Insets show ink-based surface grating (top) and mirror objective (bottom). Scale bars = 5 μm and 2 cm. (b) Holographic surface grating having a tilt angle of *θ* = 6°. Scale bar = 10 μm. (c) Grating height (nm) as a function of the width (nm) (physical width of the ink grating) for a tilt angle, *θ* = 6°. (d–g) Grating spacing as a function of structural parameters: (d) tilt angle (*θ*), (e) laser energy (*E*), (f) distance between the laser tip and sample, and (g) height variation.

The holographic patterning was performed with a single pulsed nanosecond laser (210 mJ) using in-line Denisyuk reflection mode. Here, a 532 nm laser formed standing waves to ablate permanent black ink. In Denisyuk reflection holography recording, the expanded beam passes through an ink-coated glass substrate, and is then reflected back from an object (*i.e.* plane mirror) (Fig. 1a). The object was placed normal to the incident beam and aligned with the surface plane. The ink-coated glass plate (sample) was placed over the object and the sample tilt angle (5°–35°) was varied from the surface plane. The standing wave was produced due to the interference from the incident and reflected laser beams creating constructive and destructive regions (ESI, Fig. S2†). The superposing of waves produced maximum intensity regions to ablate the ink layer. Therefore, ink-based surface patterning was achieved with periodically ablated regions. The geometry of the nanopatterned structures is dependent on the laser properties (ablation energy, wavelength, beam size) and experimental parameters (distance, height, and tilt angle variations). Sample preparation of the ink recording medium was based on the following steps: (i) the glass plates were cleaned with acetone, (ii) the plate was left to settle for 1 min to dry, black permanent ink was diluted with ethanol (100%) (1 : 1, v/v) and was spin coated (Chemat Technology, KW 4A) on the glass substrates to prepare the recording medium, (iii) spinning was performed at 1000 rpm for 60 s, followed by a frequency of 2100 rpm for 30 s, (iv) the plate was left for another 5 min to dry at 24 °C, and (v) ink-coated slides were exposed to single pulses of laser beam (532 nm) for holographic surface patterning (ESI,† ‘Sample preparation’). The preparation of the recording medium was completed within a few minutes. The absorption spectra of the prepared ink-samples showed broadband absorption peaks (ESI† ‘Laser ablation, interference and light absorption’). The absorption spectra cover the entire visible spectrum and some parts of the infrared range (400–1100 nm). Therefore, different laser wavelengths can ablate black-ink to create nanopatterns on the sample surface.

### 2.2. Ink-based diffraction gratings

To demonstrate ink-based planar optical devices, firstly parallel diffraction gratings were fabricated by Denisyuk reflection holography. Fig. 1a shows the experimental setup for producing the ink grating. Fig. 1b illustrates an environmental scanning electron microscopy (ESEM) image of the ink grating produced at a tilt angle of *θ* = 6°. Fig. 1c shows the grating width *versus* height variation for the tilt angle, *θ* = 6°. The grating spacing was ∼2.6 μm and was in agreement with computational modeling. The patterned grating structure was uniform to diffract light with high efficiency. The characteristics of the ink surface grating spacing depend on the incident laser wavelength (*λ*), energy (*E*), and structural parameters: distance between the laser tip and sample (*d*), height between the sample and the object (*h*), and the angle (*θ*) between the object and sample. The ink contains light-absorbing pigments which influences the surface patterning. The direct relationship between the grating spacing (*Λ*), laser energy (*E*) and distance between laser beam, sample and object (*d* and *h*) has not been reported previously. Therefore, an experimental study has been performed to understand their relationship and influence on the optimization of grating patterning.

Fig. 1c–f show the experimental results for the grating spacing as a function of the tilt angle (*θ*), energy (*E*), distance (*d*), and height (*h*) variation. The grating distance increased as the energy increased at a constant distance (*d* = 2.1 cm), height (*h* = 1.2 cm), and tilt angle (*θ* = 15°). The ink-surface located at the bright fringes of the interference patterns was ablated and removed. A surface grating was formed due to the ablated and non-ablated regions on the ink surface. For the single pulse ablation process, the exposure time was the same as the pulse length.^22^ A higher laser energy increased localized heat in the light-absorbing ink layer, which underwent a photothermal evaporation and ejection process. Thermal diffusion also increased with laser energy. Therefore, the grating spacing increased due to larger ablated regions as the laser energy increased. Fig. 1e–g show a linear fit curve as a function of the laser energy and exposure geometry.

**Fig. 2:**
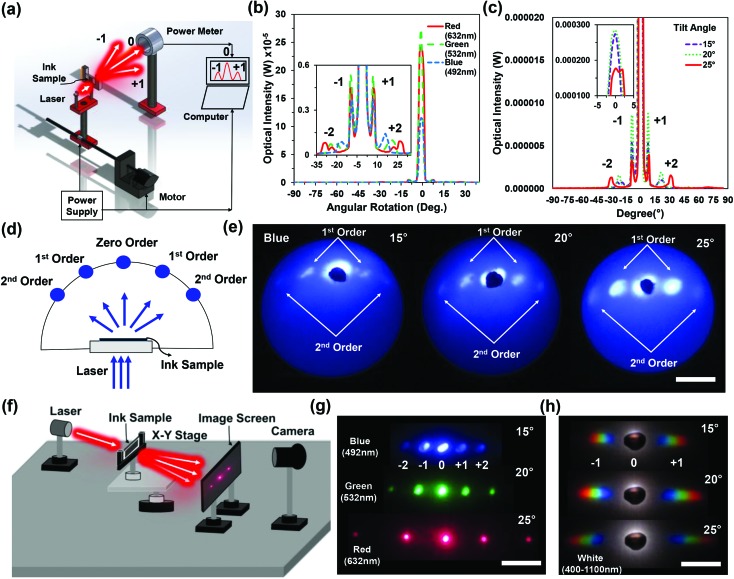
Optical characterization of the ink-based holographic gratings. (a–e) Angle-resolved measurements of the surface gratings. (a) Experimental setup for diffraction intensity measurements. (b) Optical intensity as a function of the rotation angle at a fixed tilt angle (*θ* = 15°) and at different laser wavelengths (632, 532, and 492 nm). (c) Optical intensity as a function of the rotation angle with variation of the tilt angle (*θ* = 15°, 20°, and 30°) at a fixed wavelength (632 nm). (d) Schematic of experimental setup for far-field diffraction pattern measurements with a hemispherical boundary. (e) Diffraction pattern with fabricated ink grating with tilt angle variation in a hemisphere. Scale bar = 3 cm. (f) Schematic of the experimental setup for the far-field diffraction pattern measurements on an image screen. Scale bar = 3 cm. (g) Diffraction patterns with fabricated ink grating with tilt angle and wavelength variation of laser wavelength. Scale bar = 3 cm. (h) Diffraction patterns with broadband light for the fabricated ink gratings at varying tilt angles. Scale bar = 3 cm.

The optical characterization of the fabricated ink sample was carried out with angle-resolved measurements (Fig. 2a). Red, green, and blue laser beams as well as broadband light were used to illuminate the fabricated gratings. The sample was positioned perpendicular to the reader laser beams through an *X*–*Y* rotation stage. The stage was supported with a stepper motor and rotated 180° in the horizontal plane (1° step size). The measurement data were recorded with an optical power meter placed at the 0° position. The diffraction intensity was measured as a function of rotation angle for red (632 nm, 4.46 mW), green (532 nm, 4.65 mW) and blue (492 nm, 2.6 mW) laser light (Fig. 2b). The diffraction intensity increased as the laser wavelength increased. Maximum diffraction intensity was found for the green beam and minimum for the blue beam. Analogous results were found with simulated results (ESI† ‘Simulation of diffraction grating’). Computational modeling was performed with the finite element method (FEM).^23^,^24^ Fig. 2c shows the diffraction intensity as a function of the rotation angle plotted for three different samples with tilt angle variations (*θ* = 15°, 20°, and 30°).

Maximum diffraction intensity in first order was at the 20° recording tilt angle of the sample. Diffracted light angles increased as the tilt angle increased. This was due to the decrease in grating spacing at higher tilt angles and light diffracted to larger spots, which was also confirmed by simulation results (ESI† ‘Simulation of diffraction grating’). Diffraction efficiency (DE) was measured as the ratio between diffracted (*I*_diff_) and incident (*I*_in_) laser intensity, DE = (*I*_diff_/*I*_in_) × 100%.^10^ Blue light showed maximum DE (41.53%) as compared with red (31.5%) and blue (30.2%) beams. Similarly, for the red beam, a larger tilt angle, 25° (46.9%) showed maximum DE as compared with smaller tilt angles, 20° (30.5%) and 15° (27.1%), respectively.

Optical characterization of the fabricated grating was carried out with far-field diffraction experiments. Fig. 2d shows the schematic of the experimental setup with a hemispherical boundary. The sample was illuminated normally from the bottom. Well-ordered diffraction patterns were observed on the hemispherical surface as the sample was illuminated with a green laser beam (Fig. 2e). The distance between higher orders and the zero order increased for gratings having large tilt angles. Larger tilt angles produced larger grating spacing (*Λ*) between the ablated and non-ablated ink surface. Therefore, light diffraction was increased as the tilt angle increased. Fig. 2f shows the schematic of another far-field diffraction pattern experiment. Here, the sample was fixed and the *XY*-stage was adjusted to vary the laser illumination. An image screen was used to collect the far-field diffraction patterns, which were imaged with a digital camera. The far-field diffraction patterns with varying tilt angles and laser wavelengths are shown in Fig. 2g. The distance between the zero order and higher orders increased as both the tilt angle and laser wavelength increased. As the wavelength of the laser light increased, the grating diffracted light to the larger angles, obeying Bragg's law.^8^ The diffraction properties of the fabricated sample were also tested with broadband light, producing a rainbow pattern in the far field (Fig. 2h). The distance between the rainbow pattern and zero-order specular reflection increased as the sample tilt angle was increased. Therefore, these experiments demonstrated that the ink grating efficiently diffracted the monochromatic (red, green, and blue) and broadband light.

### 2.3. 2D ink patterns

**Fig. 3:**
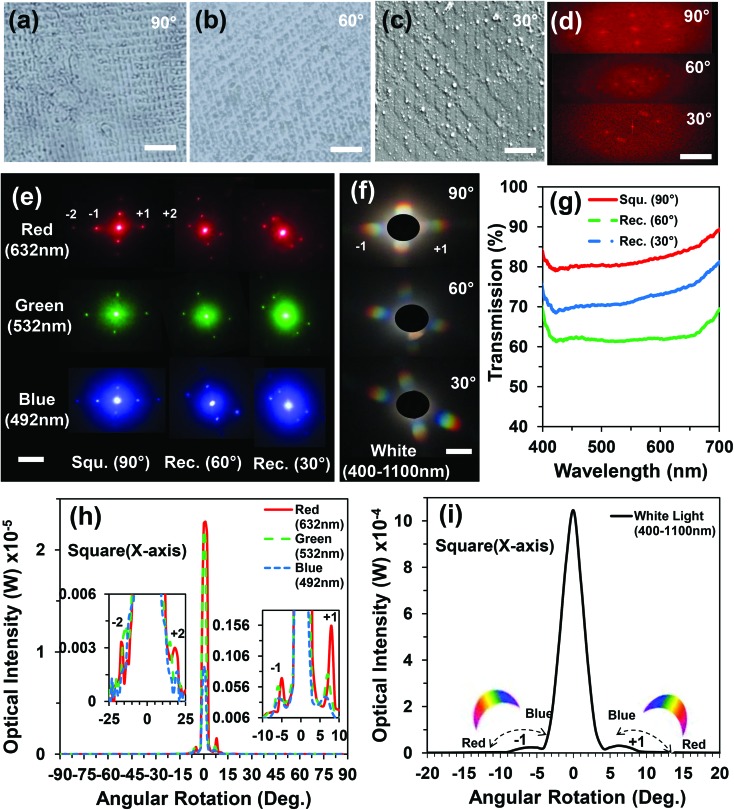
2D ink patterns fabricated by multiple laser pulses in Denisyuk reflection holography. (a–c) Square (90°), and rectangular (60° and 30°) structures. Scale bars = 5 and 2.5 μm. (d) FFT simulation of the fabricated 2D patterns. Scale bar = 25 μm. (e and f) Far-field diffraction patterns produced with red, green, blue and broadband light on an image screen. Scale bar = 3 cm. (g) Transmission power as a function of the wavelength for the square and rectangular 2D patterns. (h and i) Optical intensity as a function of the rotation angle for the square 2D pattern along the *x*-axis with variation in the readout wavelength and broadband light.

2D ink patterns were also fabricated using a modified experimental setup similar to the configuration shown in Fig. 1a. The 2D patterns were fabricated by exposing the recording medium (*i.e.* ink) to the laser pulses twice. After a single beam exposure, the sample was rotated with predefined angles, and subsequently exposed to another laser pulse. The number of pulses and sample rotation angles can be varied for producing more complex/customized structures (*e.g.* triangles, rods, and rhombuses). During 2D patterning, the exposed regions of the sample were fixed, but only the rotation angle was varied over 1 cm^2^. Fig. 3a–c show microscopy images of the fabricated 2D ink patterns fabricated with a rotation angle variation of *θ* = 90°, 60° and 30° for the second exposure. 2D patterns consisted of square (90°) and rectangular (60° and 30°) microstructures on the ink surface.

Optical characterization of the fabricated 2D patterns was performed with angle-resolved measurements and far-field diffraction experiments. 2D patterns showed diffraction in the horizontal and vertical orientations at the far-field and acted as 2D diffraction gratings. The far-field patterns for the fabricated samples were also approximated by taking a fast Fourier transform (FFT) of the patterned images. Fig. 3d shows the MATLAB-generated FFT simulation of the fabricated 2D gratings. Localized diffraction orders were observed for the simulated square (90°) and rectangular (60° and 30°) geometries. Fig. 3e and f show the experimental far-field diffraction patterns captured on an image screen placed 30 cm away from the sample.

The monochromatic (red, green, and blue) and broadband light was illuminated on the ink-based gratings. Well-ordered 2D patterns were observed in the far field and independent of readout laser wavelength. Angular diffraction measurements were performed for the square ink pattern (90°) along the *x*-direction (Fig. 3g and h). The laser lights (red, green and blue) were used to illuminate the ink grating. The optical intensity measurements as a function of the rotation angle showed that the light diffraction angle from the zero-order increased as the laser wavelength increased, analogous to 1D diffraction gratings (Fig. 3g). The light diffraction with broadband illumination also showed symmetrical higher orders analogous to the monochromatic light but with larger bandwidth (Fig. 3i).

### 2.4. Ink-based optical diffusers

Optical diffusers spread or distribute focused light and produce low-intensity soft light.^25^ The scattering or the distribution properties of the optical diffuser have applications in solar concentrators, flash/head light of cars, light-emitting diodes (LEDs)/organic-LEDs (OLEDs), medical endoscopy, displays and optical imaging.^25^–^27^ Optical diffusers may distribute light in a single direction (*x* or *y*-axis), bidirection (*x*–*y* axis), or omnidirection. Diffuser fabrication is generally based on complex nanofabrication methods such as lithography or e-beam techniques.^25^ Surface texturing of glass with direct femtosecond laser based micromachining also enables creating diffusers.

**Fig. 4:**
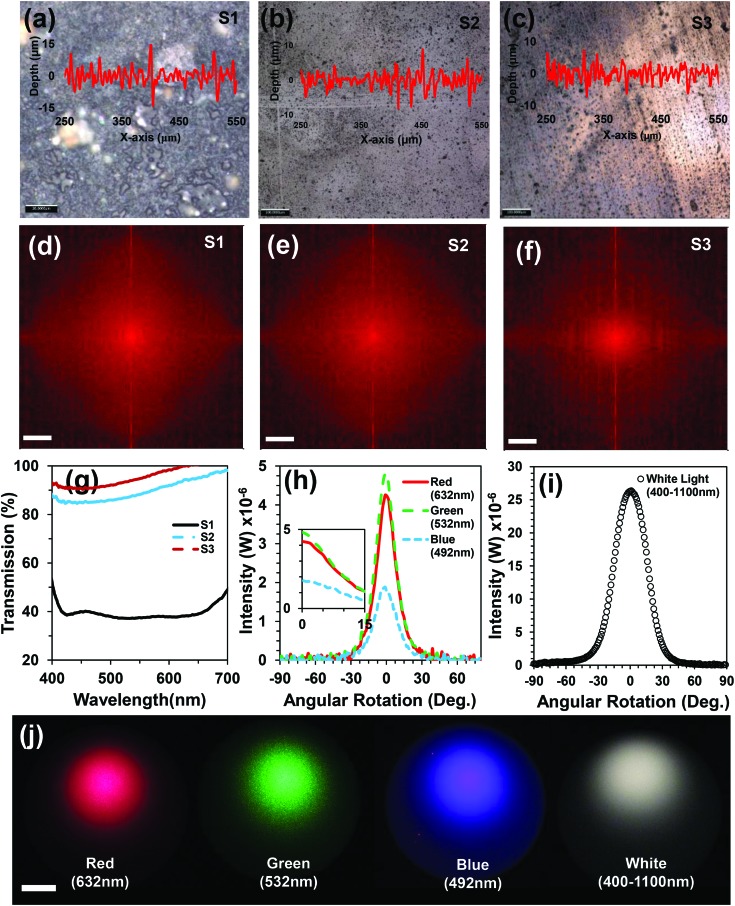
Holographic ink-based optical diffusers. (a–c) Fabricated diffuser surface with laser energy variation (90, 180 and 210 mJ). The insets show the surface roughness. Scale bars = 10 μm. (d–f) FFT simulations of the fabricated diffuser surfaces. (g) Light transmission (%) properties of the optical ink diffusers under broadband light illumination. (h and i) Angle-resolved measurements for the ink diffuser (S1) with different readout laser beams (red, green and blue) and broadband light. (j) Far-field transmitted light from the diffuser (S1) on an image screen. Scale bar = 5 cm.

Here, nanosecond reflection holography as a low-cost, simple, and rapid method was used to create optical diffusers. The ink diffuser fabrication method was similar to the diffraction grating fabrication, but a glass diffuser surface was used as an object in Denisyuk reflection mode. Fig. 4a–c show the microscopy images of the fabricated ink-based diffusers with 90, 180 and 210 mJ laser energies. As the laser power increased, most of the ink area was ablated. The surface topology was used to measure the deviation of surface roughness. Insets in Fig. 4a–c show that the surface roughness decreased as the laser power increased.

A FFT simulation was performed to quantify the relationship between the microstructure roughness and light spread from the ink diffuser (Fig. 4d–f). As the laser power increased, the light-spread area decreased and more light concentered in the central region of the simulated geometry. Light transmission and diffusion angle are two main parameters to characterize the properties of an optical diffuser. Light transmission properties of the diffuser samples were measured with respect to a glass substrate without ink (100% transmission). Fig. 4g shows transmission (%) for the illumination of broadband light on the ink-based diffuser samples. This experiment was performed with an optical microscope in transmission mode for broadband light with normal illumination on samples. As the laser power increased, the light transmission (%) also increased.

The ink-based diffuser (S1) was characterized to measure the diffusion capability as a function of the rotation angle (Fig. 4h and i). The diffusion angle of the transmitted light from the ink-based diffuser (S1) was measured with red, green, blue, and broadband beams. Green light diffused at the largest intensity and diffusion angle as compared to the red and blue beams (Fig. 4h). This may be due to surface texturing performed with the green beam. Optical diffusion was also valid for broadband light. The diffusion angle for the ink-based diffuser was ∼120°, which was dependent on laser energy and surface thickness. A far-field projection experiment was performed with a hemi-spherical surface (30 cm diameter) for red, green, blue, and broadband light illumination (Fig. 4j). The light diffusion area increased as the distance from the sample to the screen surface increased but the resolution decreased.

### 2.5. Ink-based Fresnel zone plate (FZP) lenses

**Fig. 5:**
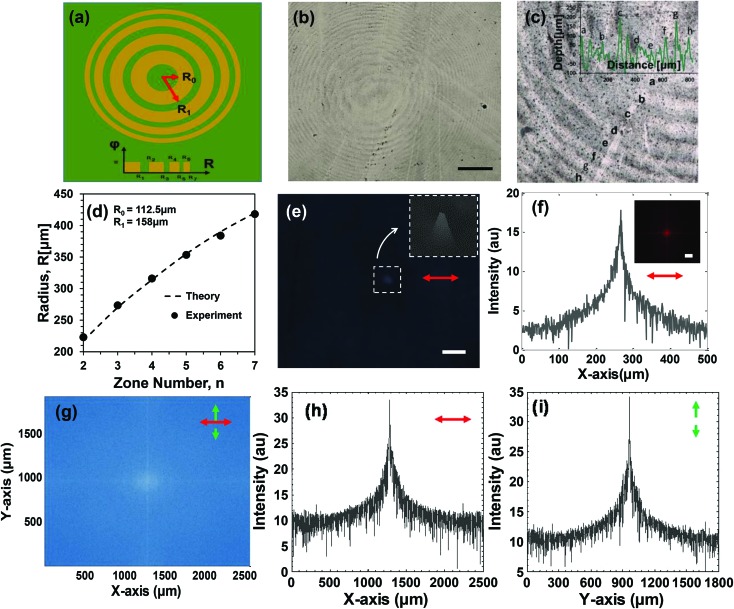
Ink-based FZP lenses. (a) Schematic of a FZP lens. (b) Bright field image of an ink-based FZP lens fabricated by Denisyuk reflection holography. Scale bar = 200 μm. (c) A magnified image of the fabricated lens. The inset shows surface patterns. Scale bar = 500 μm. (d) Radius as a function of zone number, *n* for the fabricated lens. (e) The experimental result of the focusing properties of the FZP lens for the broadband light illumination in the transmission mode of an optical microscope. (f) Focused light intensity from FFT simulation along the *x*-axis. The inset shows FFT of the focused light through a FZP pattern. (g) FFT simulation of the sample shows far-field focusing properties. (h and i) The field intensity distribution of the FFT pattern along the *x*-axis and *y*-axis of the sample.

Holographic writing can be used to fabricate ink-based flat FZP lenses. FZP is a diffractive lens consisting of a series of concentric rings, with applications in beam focusing, integrated optics, solar cells, and space navigation.^28^ Flat FZP lenses have low weight, are thinner and compact as compared with traditional curved lenses. They can also reduce distortion during imaging, which is also possible by correcting with a multilens system but require more space and cost.^29^ The fabrication of the FZP lenses was based on Denisyuk reflection holography, where the object was a concave mirror. The schematic diagram and the fabricated FZP lens are shown in Fig. 5a–c, consisting of a series of concentric rings alternating between semi-transparent and opaque regions.^30^ The focal length (*f*) of the FZP lens is:1
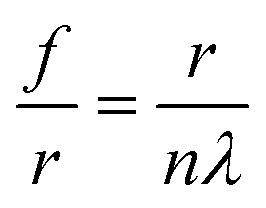
where *r*^2^ = *fnλ*, and *n* ( = 1, 2, 3, …) is an integer-value which indicates the zone number, and *λ* is the incident wavelength. In the ink-based FZP lens, the radius of the central zone was 112.5 μm. Therefore, the focal length (*f*) of the lens was at 2 cm and the wavelength at 632 nm. The majority of the incident light diffracted from the central radii. Focusing intensity depends on a number of concentric radii, where the intensity increased as the number of radii increased.

Different techniques have been reported to fabricate FZP lenses such as photolithography, stamping, and direct mechanical assembly.^29^ These approaches require pre-defined masks, expertise, and time-consuming processes. Electron beam lithography has been reported to fabricate graphene and carbon nanotube-based FZP lenses.^28^,^30^ Mask-free direct femtosecond laser writing of FZPs on buckypaper has also been reported.^29^ These techniques are limited in the number of radii (<15–20). However, in the present work, reflection holography based ink FZP lenses are reported with more than 100 radii within a few seconds. Fig. 5b and c show a fabricated ink-based FZP lens and its magnified image. As the radii increased, the width of rings decreased. Fig. 5d shows theoretical (dashes) and experimental (red dots) values along the radial direction as a function of zone numbers. As the radius increased, the width of the ring and spacing decreased, which is in agreement with the theory.

Ink-based FZP lenses were characterized to measure their focusing properties in an optical microscope for broadband light illumination in reflection mode (Fig. 5e). The inset in Fig. 5e shows a surface plot of the focused area. Simulations were also performed to compute the focused light intensity along the *x*-direction (Fig. 5f). Moreover, the far-field diffraction pattern of the fabricated FZP lens was simulated using FFT (Fig. 5g). The far-field pattern showed optical focusing at the central region of the sample. The optical intensity of the simulated far-field pattern along *x* and *y* axes also showed focusing at the center region of the FZP plate (Fig. 5h and i).

**Fig. 6:**
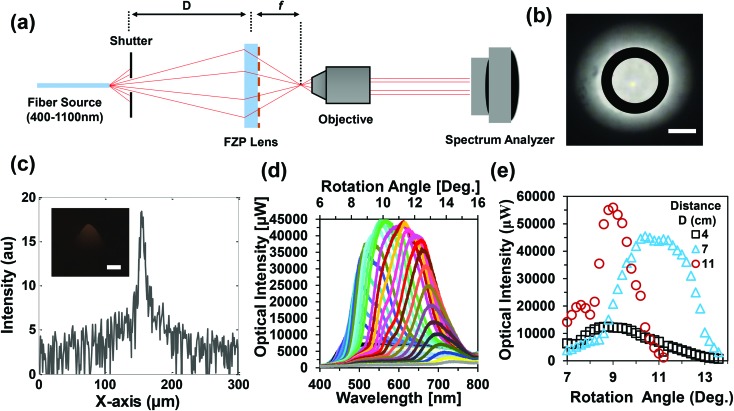
Optical properties of ink-based FZP lenses. (a) Schematic of the setup for the angle-resolved focusing measurements. (b) Focused light (black circle) on a screen for the broadband light illumination of the FZP lens. (c) Simulated focused light along the *x*-axis of the focused beam. (d) Focused light intensity as a function of wavelength and rotation angle. (e) Light intensity as a function of rotation angle for distance (*D*) variation between the spectrophotometer and the FZP lens.

Angle-resolved measurements were also performed with an ink-based FZP lens. Fig. 6a shows a schematic of the setup for angular measurements. Broadband light illuminated the ink-based FZP plate at different tilt angles and the transmitted light was measured with a spectrometer (0.2 nm resolution). Fig. 6b shows focused light on a screen at a focal point (*f*). In this case, the objective lens was replaced with an image screen and the focal point was adjusted on the screen. Simulations were also performed to analyze the focused light intensity (Fig. 6c). The inset in Fig. 6c shows the surface plot (i) and FFT (j) of the experimental focused light. Optical focusing intensity as a function of wavelength was measured for different incident angles (Fig. 6d).

Ink-based FZP lens showed different color light focusing properties with broadband light illumination at different angles. Based on the illumination angle, the FZP lens diffracted red, blue, green, and other visible light. The light diffraction properties are analogous to the conventional circular diffraction gratings. Therefore, ink-based FZP lens may work as a color-selective grating. Further analyses were performed with distance variation (*D* = 4, 7 and 11 cm) between the broadband light source and the ink-based FZP lens (Fig. 6e). For a fixed distance *D*, angle-resolved experiments were carried out to measure the maximum focused light intensity. Experiments showed that the diffraction power intensity is dependent on the incident wavelength and illumination angle. For smaller distances, the light focused sharply with high intensity as compared to the larger distances. This property has applications in focus distance adjustment of microscale optics.

## 3. Discussion

As a step toward ink-based diffractive optical elements, diffraction gratings, 2D patterns, FZP lenses, and optical diffusers have been fabricated. Low-cost ink-based planar optical components have been demonstrated using Denisyuk reflection holography. Black permanent ink served as a recording medium for laser ablation of the gratings with uniformity. The laser ablation process is compatible with black permanent ink. Laser ablation using a 532 nm beam was not possible with red and green permanent ink to achieve well-defined nanostructures. This is because the black ink absorption spectrum (ESI, Fig. S2d†) covers the visible spectrum and the interaction with 532 nm (ns) laser produces nanoscale gratings. During sample preparation, spin coating was performed at 1000 rpm for 1 min, followed by a frequency of 2100 rpm for 30 s for the thin ink layers (ESI,† ‘Sample preparation’). However, 400, 600 and 900 rpm have also been used and the laser ablation works efficiently with different ink thicknesses (ESI, Fig. S2d†). The ablation process also works efficiently with rough surfaces. However, the thick and non-uniform surface layer may reduce diffraction efficiency or light diffusion of the desired nanopatterns and require higher laser energy during the ablation process.

Holographic approaches with black permanent ink and optical characterization have been reported previously to demonstrate ink-based diffraction gratings.^7^,^12^ The diffraction properties of the 1D ink-based grating were reported and experiments followed with computational modeling. However, ink-based other optical devices (except diffraction grating) have not been reported previously. In this work, the effects of the structural parameter variation (like energy, distances (*d*), height (*h*) and angle (*θ*)) have been shown on patterning structures. These parameters influenced the geometry and optical properties of the patterned structures. The fabricated ink-based gratings may be configured to work at ultraviolet (UV) or infrared (IR) regimes. 2D ink-based gratings were patterned with square (90°) and triangular (60° and 30°) arrangement and their optical light trapping properties may find potential applications in solar cells. The far-field images of the 2D patterns with reader laser beams showed the encrypted nanostructures during recording which were not visible to the eye. Therefore, 2D patterns may be used to record optical binary data, which can be recovered from optical readouts. Different materials can be used for these gratings to expand the existing capabilities to rewritable systems to enable information multiplexing and secure data encoding.

In the present work, ink-based optical diffusers were created using Denisyuk reflection holography. Optical diffusers consisting of other materials (mostly glass or coated glass with gold, silver, photopolymers and crystals) were reported previously.^31^,^32^ The reflection holography method was used to fabricate ink-based flat FZP lenses. Dielectric ink-based lenses may find potential applications for sub-wavelength focusing and high resolution imaging.^33^ Therefore, the optical performance of the ink-based optical devices (*e.g.*, 1/2*D* gratings, optical diffusers and the FZP lens) requires further improvement. These elements have also been fabricated by two-beam or multiple-beam interferometry or e-beam writing that are complex and costly approaches.^30^,^34^ High resolution nanopatterning can be achieved by increasing the exposure tilt angle during laser ablation. A single laser exposure covers ∼1 cm^2^ ablated area (ESI, Fig. S2†) and the size of this area can be increased by varying the distance between the laser beam and the sample holder (Fig. 1f and g). Multiple exposures were required to cover a larger ablation area. A motorised *x*–*y* stage with 360° rotation is required for the precise angle variation and multiple exposures with high precision. The resolution of the reflection holographic is dependent on the exposure angle in Denisyuk reflection holography. The resolution can be enhanced by increasing the exposure tilt angle with 2D patterns. A higher resolution can be achieved with other nanofabrication techniques (e-beam or etching).^35^–^37^ However, holographic laser patterning is fast, eliminates complicated and tedious pre-/post-treatments for the sample preparation, cost-effective, and does not require high technical expertise.

The performance (surface uniformity, roughness, depth) of the ink-based optical elements produced in the present work is lower as compared to metal and glass optical devices. However, DE measurements for the ink-based optical devices are comparable to the performances of these elements made using other rapid techniques and materials.^38^ However, the holographic writing approach to fabricate diffractive optical components shows that ink can be a promising material for low-cost optical devices. A metal-ink based direct writing holography process has been recently reported through scanning transmission electron microscopy (STEM).^39^ However, holographic ink-based planar optical device fabrication simplifies and reduces processing steps and does not require specialization on chemical processing of the sample preparation, and offers mass manufacturing at low cost.

## 4. Conclusion

Ink-based diffractive optical components can be used to tailor light propagation, dispersion and wavefront shaping at the sub-wavelength scale. The presented holography method may provide implementation of any desired geometries free from optics design with advanced functionalities. Ink-based optical devices may enable optoelectronic systems including lab-on-a-chip devices. It is anticipated that ink-based planar optical devices will find applications in nanoscale optical systems, tunable endoscopy devices, and printable optics.

## Supplementary Material

Supplementary information
