# Supplementary material for: Printable ink lenses, diffusers, and 2D gratings
Source: Nanoscale. 2016 Nov 23;9(1):266–76. doi: 10.1039/c6nr07841a (PMC6089092; doi:10.1039/c6nr07841a)
Supplement: Supplementary file 1 [file NR-009-C6NR07841A-s001.pdf]

Electronic Supplementary Material (ESI) for Nanoscale.
This journal is © The Royal Society of Chemistry 2016

SUPPORTING INFORMATION

Printable Ink Lenses, Diffusers, and 2D Gratings

Rajib Ahmed,[1] Ali K. Yetisen,[2,3] Anthony El Khoury,[1] and Haider Butt[1,*]

[1]Microengineering and Nanotechnology Laboratory, School of Mechanical Engineering, University of Birmingham, Birmingham B15 2TT, United Kingdom

[2]Harvard Medical School and Wellman Center for Photomedicine, Massachusetts General Hospital, 50 Blossom Street, Boston, Massachusetts 02114, USA

[3]Harvard-MIT Division of Health Sciences and Technology, Massachusetts Institute of Technology, Cambridge, MA, 02139, USA

*Email: h.butt@bham.ac.uk Tel.: +441214158623

Methods

Materials and Equipment. Glass was used as a substrate and its surface was coated with black permanent ink (Staedtler, Germany) diluted in ethanol. An Nd:YAG laser was operated at 532 nm and 210 mJ. A spectrometer (resolution ~0.1-100 nm FWHM) and a broadband light source (450-1100 nm) were purchased from Ocean Optics for optical measurements. COMSOL Multiphysics (v5.1), and MATLAB (MathWorks, v8.1) were used for finite element simulations and data processing, respectively.

Sample Preparation

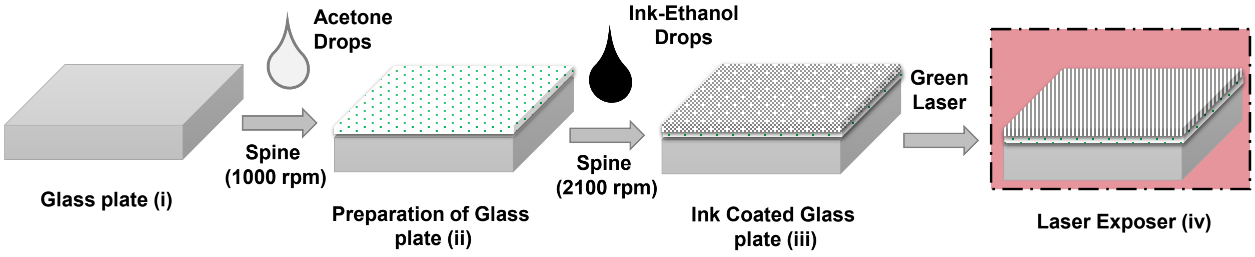

Figure S1. Sample Preparation

Laser Ablation, Interference, and Light Absorption. Figure S2a shows laser ablation (90 mJ) of an ink layer to create a diffraction grating. There were three zones of patterned region after ablation: ablated (A1), molten (A2) and non-ablated (A3) zones. Figure S2b shows a magnified view of the ablated region and surface roughness measurements.

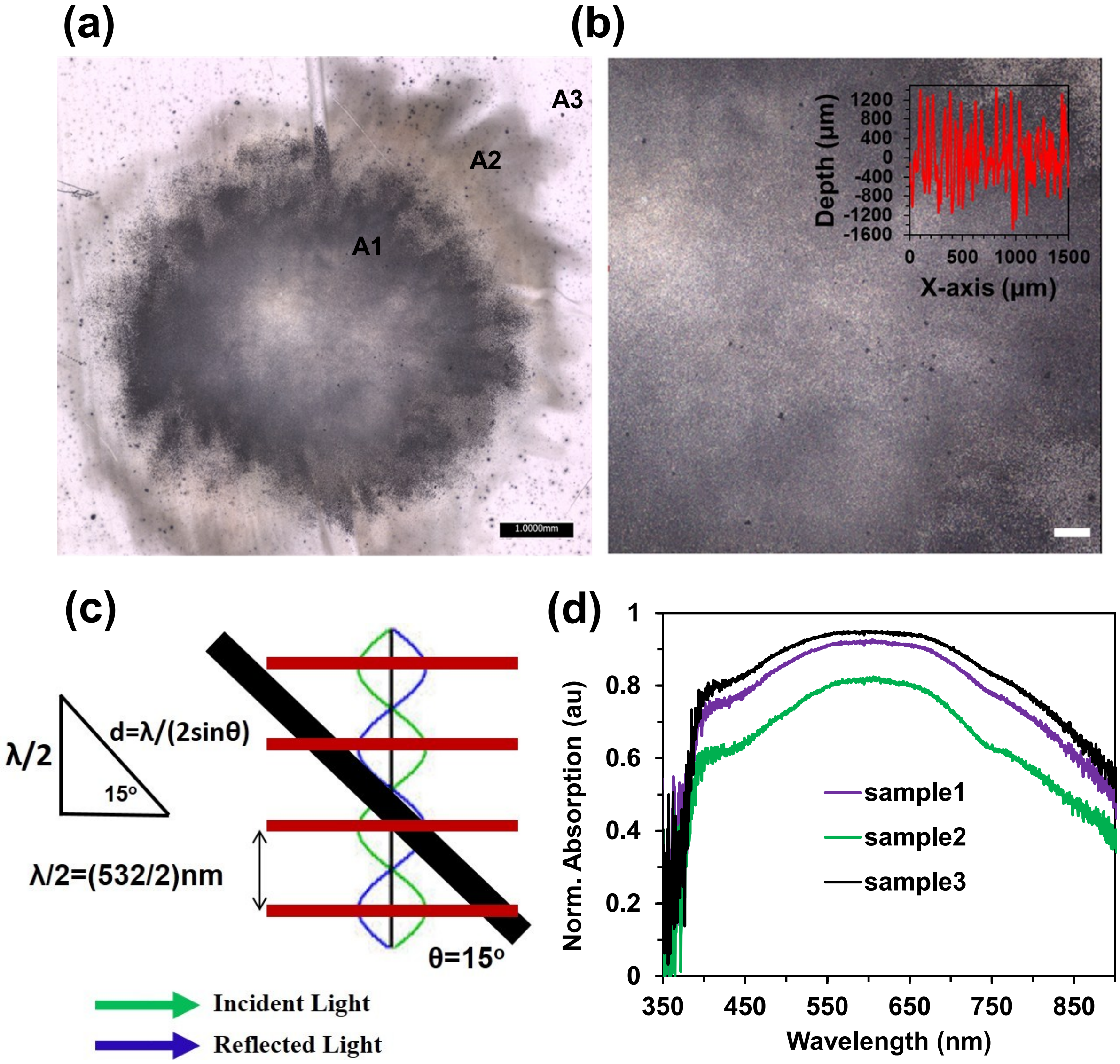

Figure S2. Laser ablation of ink in Denisyuk reflection mode. (a) Ablated ink surface on glass substrate. Scale bar= 1 mm. (b) Magnified view of the ablated zone. Scale bar= 200 µm. (c) Conceptual diagram for laser interference in the ink-based recording medium. (d) Absorption spectrum of the ink.

The laser-induced surface patterning process in the ink film can be explained by the interference phenomenon.[1] An arbitrary incident wave (reference beam) is:

$$E_i = A\cos 2\pi(\nu t - \frac{x}{\lambda}) \quad \text{(Eq. S1)}$$

The reflected wave (object beam) from the mirror in the opposite direction is:

$$E_r = A\cos 2\pi(\nu t + \frac{x}{\lambda}) \quad \text{(Eq. S2)}$$

The interference wave formed in the ink film is:

$$E = E_i + E_r = A\cos 2\pi(\nu t - \frac{x}{\lambda}) + A\cos 2\pi(\nu t + \frac{x}{\lambda}) = \left|2A\cos 2\pi(\frac{x}{\lambda})\right|\cos 2\pi\nu t \quad \text{(Eq. S3)}$$

The minimum intensity of the interference wave occurs at out of phase condition (waves are maximum in opposite direction, red bars in Figure S2c) and create destructive interference regions (nodes in the standing wave). Therefore, surface patterns formed in the ink film consist of ablated and non-ablated regions. Patterned structure also depends on laser properties and structural parameters. Additionally, the absorption spectrum of the ink films was measured (Figure S2d). Three samples were prepared and they had differences in the absorption due to thickness variation. However, the absorption range (400-900 nm) of the ink allows ablation with red (632 nm), green (532 nm) and blue (492 nm) laser beams.

Simulation of Ink-Based Diffraction Grating. The diffraction simulations of the ink grating were performed with finite element based COMSOL Multiphysics software.[2, 3] The structure of the grating was considered as hemispherical with a complex refractive index (n=1.6+1.6j). Figure S3a shows a schematic of the simulation environment. The grating spacing was $\Lambda = 1027$ nm for the green wavelength (λ=532 nm) with a tilt angle, θ=20°, according to the. Bragg's law (spacing, Λ=λ/2sinΘ, where λ is the wavelength, and Θ is the tilt angle from the surface plane).

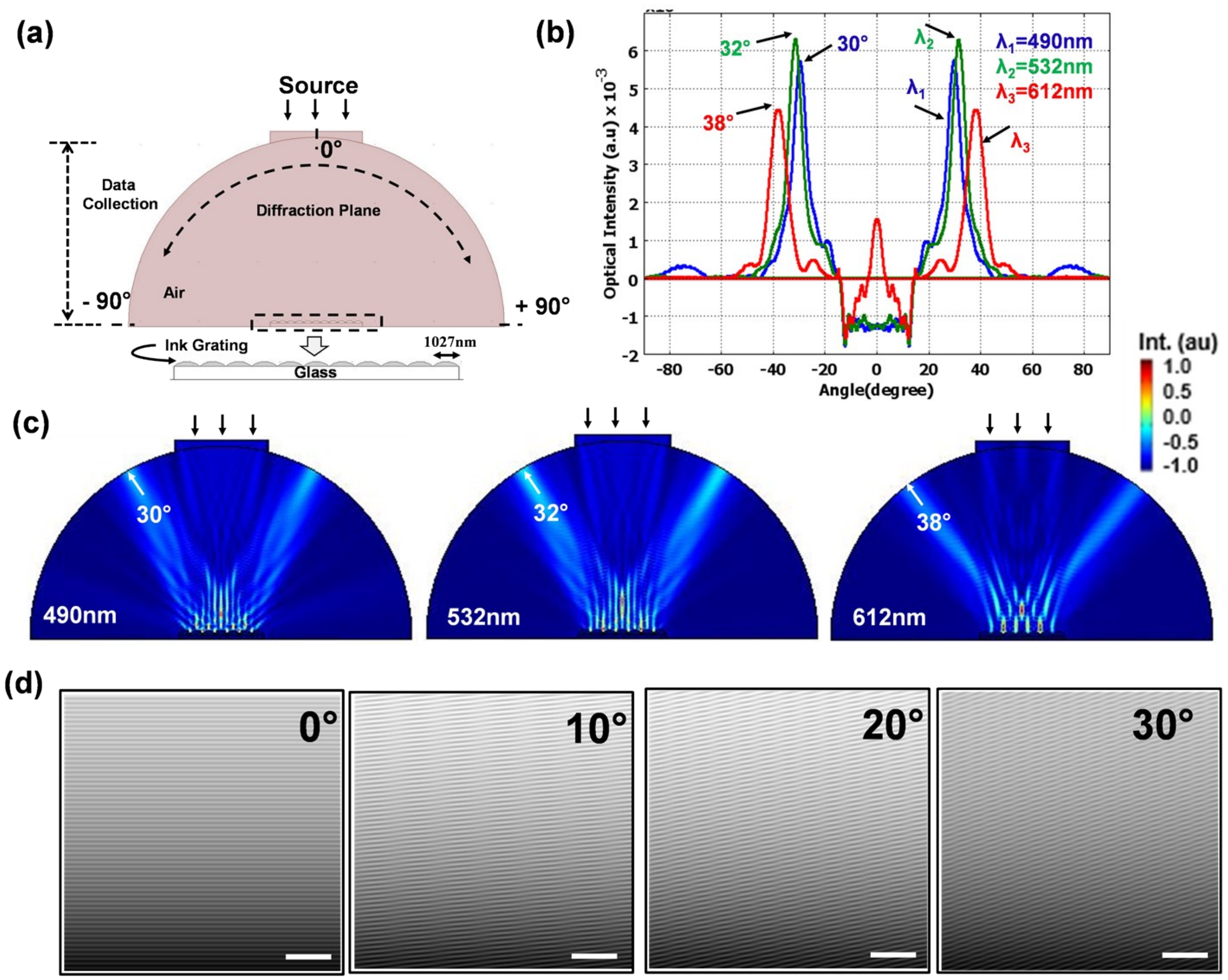

Figure S3. Computation of the ink-based holographic surface grating. (a) Simulation diagram for the diffraction from the ink-based grating. (b) Simulated optical intensity for incident laser wavelengths (red, green, and blue). (c) E-field intensity distribution for incident blue (490 nm), green (532 nm), and red (632 nm) laser wavelengths. Scale bar = 1 µm.

Figure S3b shows the diffraction intensity as a function of the incident wavelength (red, green, and blue). The light diffracted to the larger angles as the reader wavelength increased. The red light diffracted to maximum (38°) and minimum (30°) angles for the blue light, obeying the grating formula. The maximum optical intensity was obtained with green light (32°). This is due

to the reason that green light (λ=532 nm) was considered during modeling for the grating spacing, Λ= 1027 nm with a tilt angle, θ=20°. Figure S3c shows the electric field intensity distribution with incident wavelength variation (Figure S3b).

To evaluate the optical phenomenon during grating formation, computation modeling has been performed as a function of inference angle (tilt) with MATLAB (Figure S3d). The recording medium was considered ~30 μm for the model. The grating was formed in the ink layer due to constructive and destructive interference of incident and reflected beams as well as the light reflected from ink-air (or glass substrate) interface. The interference pattern was mainly due the superposition of the two distinct plane waves: the incident wave and the reflected wave from the plane mirror. The simulation results showed that the grating spacing decreased as the tilt angle increased from the surface plane, which was in agreement with the experiment results.

References

1. Q. Zhao, A. K. Yetisen, C. J. Anthony, W. R. Fowler, S. H. Yun and H. Butt, *Appl. Phys. Lett.*, 2015, **107**, 041115.
2. H. Butt, A. K. Yetisen, R. Ahmed, S. H. Yun and Q. Dai, *Appl.Phys. Lett.*, 2015, **106**, 121108.
3. R. Ahmed, A. A. Rifat, A. K. Yetisen, Q. Dai, S. H. Yun and H. Butt, *J. Appl. Phys.*,, 2016, **119**, 113105.
